# Endoscopic ultrasound guided fine needle aspiration versus endoscopic ultrasound guided fine needle biopsy in sampling pancreatic masses: A meta-analysis

**DOI:** 10.1097/MD.0000000000007452

**Published:** 2017-07-14

**Authors:** Jing Wang, Shulei Zhao, Yong Chen, Ruzhen Jia, Xiaohua Zhang

**Affiliations:** a Department of Infectious Diseases; b Department of Gastroenterology, Shandong Provincial Hospital Affiliated to Shandong University, Jinan, China.

**Keywords:** endoscopic ultrasound, FNA, FNB, pancreatic mass

## Abstract

**Background:**

The comparison between endoscopic ultrasound guided fine needle aspiration (EUS-FNA) and endoscopic ultrasound guided fine needle biopsy (EUS-FNB) for the diagnosis of pancreatic masses is still controversial. Many factors can affect the final results.

**Methods:**

Databases, such as PubMed, EMBASE, Cochrane Library, and Science Citation Index updated from 2000 to 2016 were searched to include eligible articles. In the meta-analysis, the main outcome measurements were the diagnostic accuracy, number of needle passes, specimen adequacy, the rate of complications, and technical success.

**Results:**

Eight randomized controlled trials (RCTs) were identified, and a total of 921 cases were included in the meta-analysis. The diagnostic accuracy was not significantly different between the FNA and FNB groups. The specimen adequacy was higher in the FNB group compared with the FNA group. The number of needle passes to obtain sufficient tissue was lower in the FNB group. The rate of adverse events and technical success did not significantly differ between the 2 groups. But, the forest plot showed a trend toward lower technical success rate and a trend toward higher diagnostic accuracy in the FNB group, compared with FNA.

**Conclusion:**

We provide the evidence that FNB is comparable to FNA in terms of diagnostic accuracy, adverse events, and technical success. FNB gives higher specimen adequacy than that of FNA, despite performance of fewer needle passes.

## 1 Introduction

Some epidemiologic surveys have reported that the 5-year survival rate of pancreatic cancer is below 5%.^[1,2]^ It is difficult to accurately diagnose pancreatic lesions because of the late onset of symptoms. The diagnosis of pancreatic lesions was significantly improved because of the application of endoscopic ultrasonography. Endoscopic ultrasound guided fine needle aspiration (EUS-FNA) is the standard diagnostic tool to obtain tissue for the accurate diagnosis of pancreatic masses since 1990s.^[3–5]^ The reported results of pancreatic EUS-FNA vary in the range of 84% to 92.9% for diagnostic accuracy.^[6–8]^ Hewitt et al^[9]^ reported that the sensitivity and specificity of EUS-FNA pancreatic neoplasms were 85% and 98%.

EUS-FNA often only provides cytologic samples for diagnosis. Certain neoplasms, such as lymphoma and stromal tumors, require histological specimen to assess both the tissue architecture and cell morphology. In order to overcome the limitations, a new fine needle biopsy device with a reverse bevel at the tip to collect a core sample (core needle) has been designed. A meta-analysis conducted by Yang et al^[10]^ reported that endoscopic ultrasound guided fine needle biopsy (EUS-FNB) is a reliable diagnostic tool for solid pancreatic masses with good diagnostic accuracy and should be especially considered for pathology where histologic morphology is preferred for diagnosis. The sensitivity and specificity of EUS-FNB for pancreatic neoplasms were 84% and 98%, respectively.

The results of the studies which have compared EUS-FNA and EUS-FNB for pancreatic lesions are not completely consistent, and there has been no meta-analysis that could evaluate the differences with greater statistical power. We conducted a meta-analysis to compare the efficacy and safety of EUS-FNA to EUS-FNB in sampling pancreatic masses.

## 2 Materials and methods

### 2.1 Data sources and searches

We searched databases including PubMed, EMBASE, the Cochrane Library, and Science Citation Index updated from January 2000 to June 2016 to identify related articles, without language restriction, which compared EUS-FNA and EUS-FNB. All bibliographies were indentified in the reference lists. The searching terms were used: “FNA,” “FNB or core needle,” and “pancreatic.” Major proceedings of international meetings (such as Digestive Disease Week, Asian Pacific Digestive Week, and so on) were also hand-searched.

### 2.2 Study selection

Inclusion criteria: patients with suspected pancreatic mass; study was conducted as a randomized control trials (RCTs) comparing EUS-FNA and EUS-FNB for pancreatic masses; final diagnosis was resolved; written in English; and provided sufficient data to extract diagnostic results such as the diagnostic accuracy, number of needle passes, specimen adequacy, adverse events, and technical success.

Exclusion criteria: case report, comments, reviews, or guideline articles; non-RCT studies; and insufficient data.

### 2.3 Data extraction

Data were extracted by 1 investigator and confirmed by the other according to a predefined data extraction form. Disagreements were resolved by consultation with a 3rd investigator. The following data were collected: year of publication, first author, country, duration, mean age, sex, tumor size, diagnostic accuracy, number of needle passes, specimen adequacy, adverse events, and technical success.

### 2.4 Statistical analysis

All data extracted were entered in the freeware program Review Manager (Version 5.0 for Windows, Cochrane Collaboration). The weighted mean difference was recommended for continuous data, and the odds ratio (OR) with 95% confidence intervals (CIs) was recommended for dichotomous data. Statistical heterogeneity between trials was evaluated by the chi-square test and was considered to be present when *P* less than .1. We also used *I*^2^ to assess the heterogeneity. *I*^2^ more than 50% was considered to be statistical significance. In the presence of statistical heterogeneity, heterogeneity was explored by subgroup analysis or a random-effects model. Publication bias was detected by a funnel plot, and then the symmetry of the funnel plot was confirmed by the Egger test, with a *P* value of .05.

## 3 Results

### 3.1 Study selection

**Figure 1:**
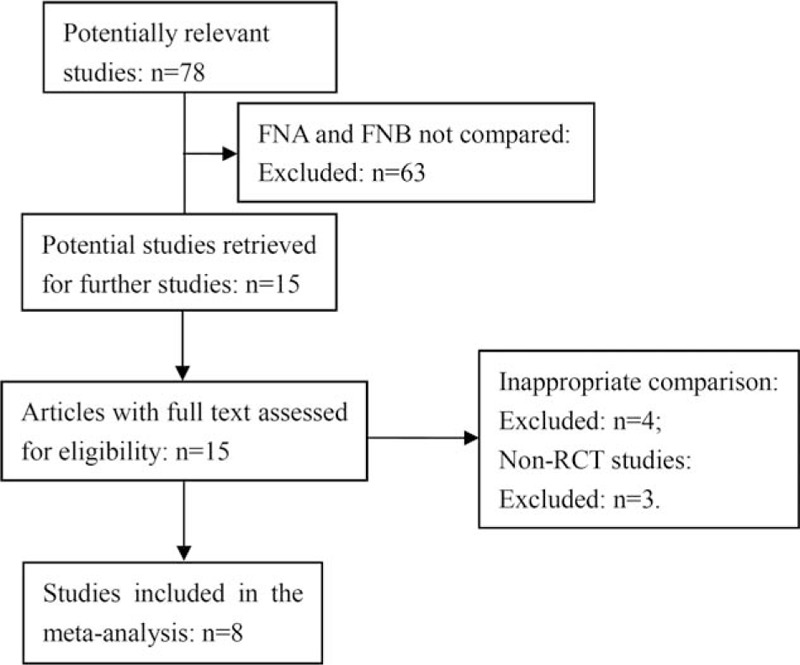
Flow diagram of trial selection.

A total of 78 potential studies were retrieved for the meta-analysis, 63 were excluded because FNA and FNB were not compared. Of the 15 articles, 4 were excluded for inappropriate comparison, 3 were excluded for non-RCTs. The remaining 8 eligible studies^[11–18]^ were chosen for further analysis (Fig. 1). A total of 921 cases were included in the meta-analysis, including 462 cases in the FNA group and 459 cases in the FNB group. All of the studies were prospective RCTs. The key characteristics of the studies are listed in Table 1.

**Table 1:**
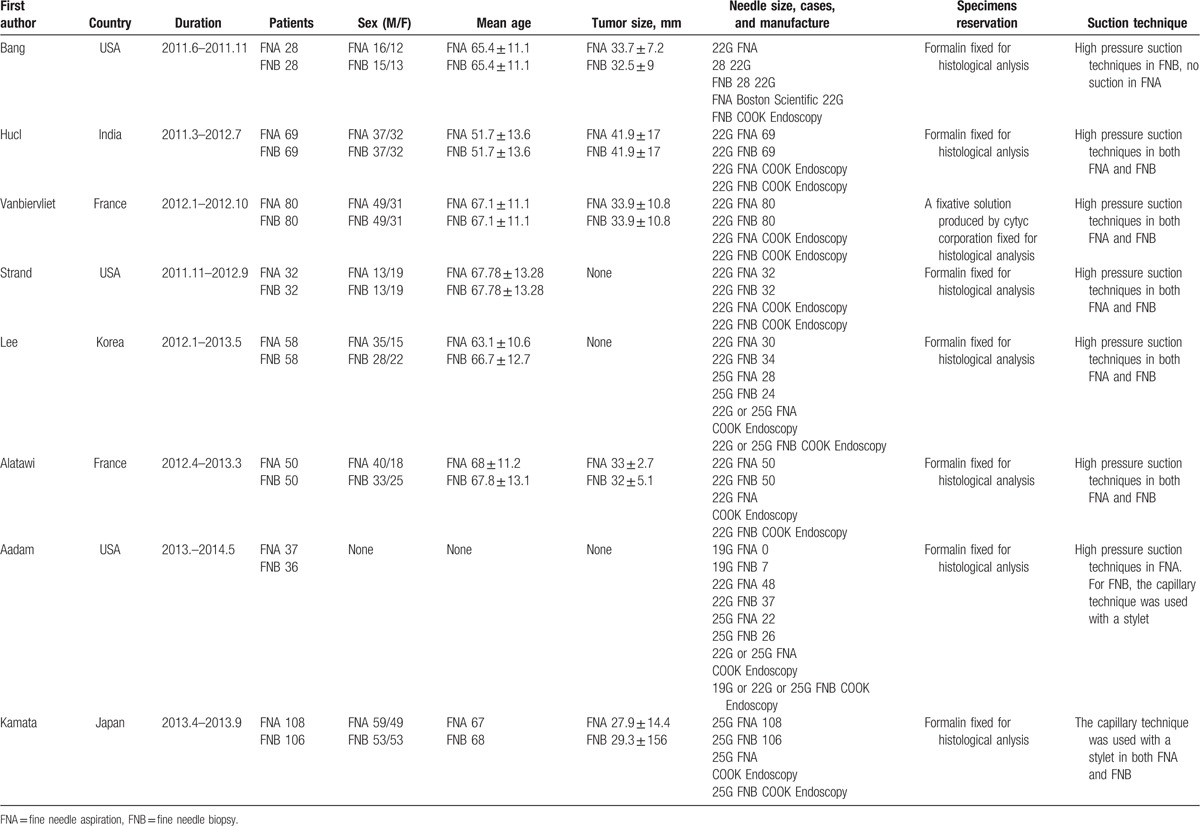
The key characteristics of the included studies.

### 3.2 Diagnostic accuracy

**Figure 2:**
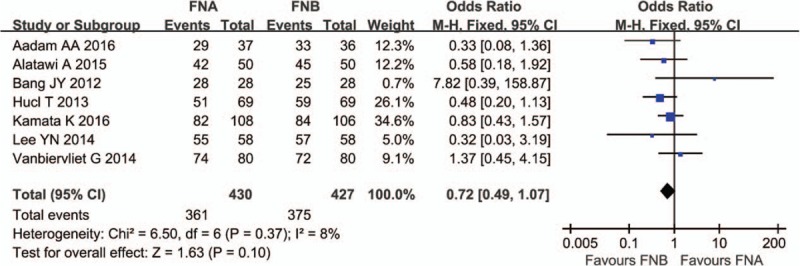
Diagnostic accuracy comparing FNA and FNB. EUS-FNA = endoscopic ultrasound guided fine needle aspiration, EUS-FNB = endoscopic ultrasound guided fine needle biopsy.

The diagnostic accuracy was reported in all of the included 8 studies.^[11–18]^ There was heterogeneity among the studies (*P* = .0001, *I*^2^ = 76%). We excluded the study from Strand et al,^[14]^ and the heterogeneity was eliminated (*P* = .37; *I*^2^ = 8%). A fixed effect model was applied. The analysis showed the diagnostic accuracy was comparable in the FNA group (361/430) and the FNB group (375/427) (OR 0.72; 95% CI, 0.49–1.07) (Fig. 2).

### 3.3 Specimen adequacy

**Figure 3:**
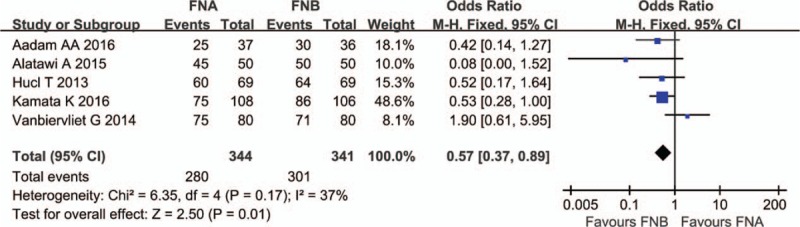
Specimen adequacy comparing FNA and FNB. FNA = fine needle aspiration, FNB = fine needle biopsy.

The specimen adequacy was reported in 5 studies.^[12,13,16–18]^ There was no heterogeneity in the studies (*P* = .17, *I*^2^ = 37%), and a fixed effect model was applied. The specimen adequacy was higher in the FNB group (301/341) compared with the FNA group (280/344) (OR 0.57; 95%CI, 0.37–0.89) (Fig. 3).

### 3.4 Number of needle passes

**Figure 4:**
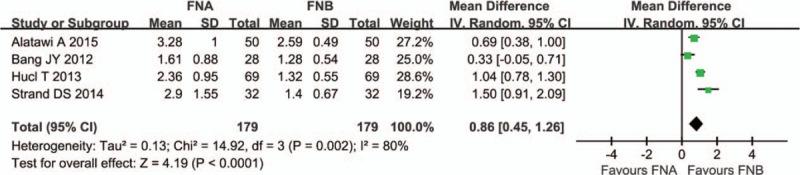
Number of needle passes comparing FNA and FNB. FNA = fine needle aspiration, FNB = fine needle biopsy.

The number of needle passes were reported in 4 studies.^[11,12,14,16]^ A random-effect model was applied because of the obvious heterogeneity (*P* = .002, *I*^2^ = 80%). The analysis showed fewer needle passes were needed in the FNB group compared with the FNA group (OR 0.86; 95%CI, 0.45–1.26) (Fig. 4).

### 3.5 Adverse events

**Figure 5:**
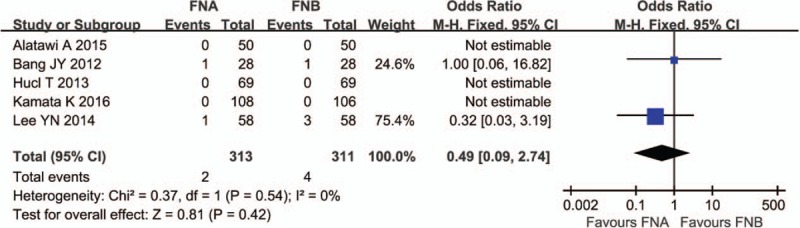
Adverse events comparing FNA and FNB. FNA = fine needle aspiration, FNB = fine needle biopsy.

The adverse events were reported in 5 studies.^[11,12,15,16,18]^ There was no heterogeneity among the studies (*P* = .54, *I*^2^ = 0%). The subsequent analysis showed that the rate of adverse events did not significantly differ between the 2 groups (OR 0.49; 95% CI, 0.09–2.74) (Fig. 5).

### 3.6 Technical success

**Figure 6:**
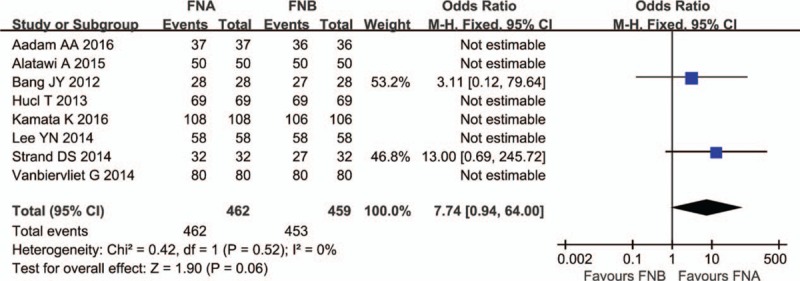
Technical success comparing FNA and FNB. FNA = fine needle aspiration, FNB = fine needle biopsy.

The technical success was reported in the present 8 studies.^[11–18]^ There was also no heterogeneity in these studies (*P* = .52, *I*^2^ = 0%), and a fixed effect model was applied. The analysis showed that there was no significant difference between the FNA group and FNB group (OR 7.74; 95% CI, 0.94–64) (Fig. 6).

### 3.7 Publication bias

We used the diagnostic accuracy as the outcome, and no publication bias was detected by funnel plot and the Egger test (*P* = .430).

## 4 Discussion

The comparison between EUS-FNA and EUS-FNB for the diagnosis of pancreatic masses is still controversial. Several factors can affect the results, such as the nature of the target, the experience of the endoscopic experts, the type of the needles, the number of needle passes, and the presence of an onsite cytologist or pathologist. Therefore, we designed the meta-analysis to systematically evaluate the 2 methods, providing evidence for the optimal technique to accurately diagnose pancreatic masses. In the present analysis, 8 RCTs were included. The results confirmed that the diagnostic accuracy was comparable in the FNA and FNB group, but there was a trend toward the core needle exhibiting higher diagnostic accuracy than the aspiration needle. If more studies could be included in the future, the diagnostic accuracy may be different between the 2 groups.

Leblanc et al^[19]^ reported that the optimal number of EUS-FNA needle passes to achieve a diagnosis ranges from 2 to 6. The number of needle passes was reported in 4 of the included studies. The analysis showed fewer needle passes were needed in the FNB group compared with the FNA group, which means an advantage of a shorter operation time, resulting in decreases in anesthesia duration, medical cost, and adverse events. The specimen adequacy was reported in 5 studies. The pool analysis showed it was higher in the FNB group compared with the FNA group. It means that the specimen adequacy using the core needle was higher than that of FNA, despite performance of fewer needle passes.

It had been reported that the adverse event rate for EUS-FNA was less than 1%.^[20]^ Yang et al reported the EUS-FNB had a comparable adverse event rate with EUS-FNA.^[10]^ Of the studies selected in this meta-analysis, only 2 studies reported adverse events after operation, including acute pancreatitis, abdominal pain, bleeding, and gastric hematoma, all patients recovered rapidly after conservative treatment. The pooled analysis showed the rate of adverse events did not significantly differ between the 2 groups.

All of the included studies have compared the technical success between the 2 groups. The results showed the rate of technical success was 100% for EUS-FNA in all studies and for EUS-FNB in 6 studies, Bang et al^[11]^ and Strand et al^[14]^ reported the success rates were 96.4% and 84.4% in the EUS-FNB group, respectively. The rate of technical success did not significantly differ between the 2 groups. But, the forest plot showed a trend toward lower technical rate of FNB, compared with FNA.

Several limitations of our study need to be considered: First, it was not possible to blind the endosonographers to the type of needle used; second, a maximum of 2 core biopsy passes were performed in Strand's article^[14]^; and third, the presence of heterogeneity cannot be eliminated when we analyze the number of needle passes.

In conclusion, we provide the evidence that EUS-FNB is comparable to EUS-FNA in terms of the diagnostic accuracy, adverse events, and technical success. Meanwhile, EUS-FNB gives fewer number of needle passes to obtain sufficient tissue and higher specimen adequacy.
